# Brain Magnetic Resonance Imaging Findings in Children after Antenatal Maternal Depression Treatment, a Longitudinal Study Built on a Pilot Randomized Controlled Trial

**DOI:** 10.3390/ijerph16101816

**Published:** 2019-05-22

**Authors:** Laura S. Bleker, Jeannette Milgrom, Donna Parker, Alan W. Gemmill, Christopher J. Holt, Alan Connelly, Huibert Burger, Tessa J. Roseboom, Susanne R. de Rooij

**Affiliations:** 1 Department of Obstetrics and Gynecology, Amsterdam UMC, University of Amsterdam, 1105 AZ Amsterdam, The Netherlands; t.j.roseboom@amc.uva.nl; 2 Amsterdam UMC, Department of Clinical Epidemiology, Biostatistics and Bioinformatics, University of Amsterdam, 1105 AZ Amsterdam, The Netherlands; s.r.derooij@amc.uva.nl; 3 Parent-Infant Research Institute, Heidelberg, VIC 3084, Australia; jeannette.milgrom@austin.org.au (J.M.); alan.gemmill@austin.org.au (A.W.G.); christopher.holt@acap.edu.au (C.J.H.); 4 Melbourne School of Psychological Sciences, University of Melbourne, VIC 3010, Australia; 5 Florey Institute of Neuroscience and Mental Health, Melbourne Brain Centre, Heidelberg, VIC 3084, Australia; donna.parker@florey.edu.au (D.P.); a.connelly@brain.org.au (A.C.); 6 Department of General Practice, Interdisciplinary Center Psychopathology and Emotion regulation (ICPE), University Medical Center Groningen, 9712 CP Groningen, The Netherlands; h.burger@umcg.nl

**Keywords:** magnetic resonance imaging, brain, cognitive therapy, embryonic and fetal development, depression, anxiety, child development

## Abstract

Antenatal depression is associated with an increased risk of offspring neuro-developmental disorders, potentially as a consequence of an altered brain development *in utero*. We hypothesized that reducing maternal depression by Cognitive Behavioral Therapy (CBT) during pregnancy may ameliorate the offspring’s brain (micro)structural outcomes. 54 pregnant women with a diagnosed clinical depression were randomly allocated to CBT or Treatment as Usual (TAU), showing moderate to large depression symptom improvements after CBT. In 16 of their children (69% boys, N(TAU) = 8, N(CBT) = 8, mean age = 5.9 years, range = 3.9–7.1 years) brain Magnetic Resonance Imaging (MRI) scans were conducted. Children from the CBT group had a thicker right lateral occipital cortex (difference: 0.13 mm, 95% CI = 0.005–0.26) and lingual gyrus (difference: 0.18 mm, 95% CI = 0.01–0.34). In the CBT group, Voxel-Based Morphometry analysis identified one cluster showing increased gray matter concentration in the right medial temporal lobe at *p* < 0.05 uncorrected, and fixel-based analysis revealed reduced fiber-bundle cross-section in the Fornix, the Optical Tract, and the Stria Terminalis at *p* < 0.01 uncorrected. However, none of the results survived correction for multiple testing. Our explorative analyses provided some indication that antenatal CBT for depression may ameliorate offspring’s brain (micro)structural outcomes, but the sample size was extremely small, and our results should be cautiously interpreted. Larger studies are warranted to confirm our preliminary conclusions that CBT for antenatal depression affects brain development in children.

## 1. Introduction

There is considerable evidence that prenatal exposure to maternal depression is associated with less favorable child outcomes, with varying directions of effects depending on the timing of the exposure, as well as the outcome measured [1,2,3,4]. Antenatal depression is a common disorder with prevalence estimates of 7.4 to 12.8% in western societies [5], and is highly comorbid with anxiety [6]. Higher levels of depression and anxiety in pregnancy have been linked to more “difficult” temperament, and more emotional and behavioral problems in children, also when postnatal confounding factors were taken into account [4,7,8,9,10].

The potential biological mechanisms mediating these associations have only recently started to be unraveled [11], with an emphasis on the insult-sensitive fetal brain [12,13]. Neonates prenatally exposed to maternal depression show altered functional connectivity, as well as lower fractional anisotropy and axonal diffusivity between the amygdala and the left temporal cortex and insula, the bilateral anterior cingulate, medial orbitofrontal and ventromedial prefrontal cortices [13,14]. Studies in children in early to late childhood report that prenatal exposure to maternal depression is associated with cortical thinning, primarily in the right superior, medial orbital, and frontal pole regions of the prefrontal cortex, as well as in temporal brain regions [15,16,17], and an increased volume of the amygdala [18].

These studies suggest that antenatal depression during pregnancy independently contributes to offspring brain development, but the observational study designs are hampered by potential confounding factors, that are both associated with antenatal depression and offspring neurodevelopment, such as maternal smoking in pregnancy [19,20]. The highest level of evidence can be obtained only by performing experimental studies in humans that include biological substrates, which, to the best of our knowledge, has not been done yet [2]. We aimed to address this gap in the literature, by conducting an explorative brain Magnetic Resonance Imaging (MRI) study, in children born to mothers who had participated in a pilot Randomized Controlled Trial (RCT) on the effectiveness of Cognitive Behavioral Therapy (CBT) for depression during their pregnancy [21]. We hypothesized that treatment of maternal antenatal depression would ameliorate the assumed effects on the offspring’s brain (micro-)structure observed in prior observational studies in children prenatally exposed to maternal depression.

## 2. Methods and Materials

### 2.1. Participants

This is a longitudinal follow-up study in children, that was built on a pilot Randomized Controlled Trial (RCT), in which their mothers had participated when they were pregnant. The pilot RCT was initially developed to investigate whether a pregnancy-adjusted Cognitive Behavioral Therapy (CBT) could reduce depression and anxiety symptoms in pregnancy, and concurrently improve infant neurodevelopmental outcomes [21]. Women ≥ 18 years and <30 weeks pregnant were recruited through screening programs at the Northern and Mercy Hospitals for Women, Melbourne, Australia, and via other health professionals and services in the public (e.g., obstetricians) and in the private sector. The Edinburgh Postnatal Depression Scale (EPDS) was used to screen women for depression. Women with a score of ≥13 on the EPDS, which is indicative of a possible antenatal depression [22], were referred for a comprehensive psychological assessment by a psychologist, using the Diagnostic and Statistical Manual of Mental Disorders (DSM)-IV criteria for a depressive disorder, or an adjustment disorder with mixed depression and anxiety [23]. Risk requiring crisis management, comorbid axis I disorders, or medical conditions that were expected to significantly affect study participation, involvement in other psychological programs, or significant difficulty with English, were the exclusion criteria [21]. The Beck Depression and Anxiety Inventories (BDI-II [24] and BAI [25] respectively) were completed by women who met DSM-IV criteria for a depressive disorder or adjustment disorder, to assess symptom scores of depression and anxiety. Consenting, eligible women were randomized to receive CBT or Treatment as Usual (TAU). The CBT program consisted of eight individually administered weekly sessions of an hour including one partner session.

TAU consisted of case-management by a midwife or general practitioner, who could refer to other specialized services if necessary. A detailed description of randomization procedures and power calculations for primary outcomes is described elsewhere [21]. At the end of the program, moderate to large reductions of depression and anxiety symptoms were seen in the CBT group compared to TAU, with more beneficial developmental infant outcomes at nine months of age in this group as well, showing better self-regulation, problem solving and stress reactivity, independent of postnatal maternal mood, compared to the infants of the TAU group [21]. For the current study, approximately six years postpartum, all children born to mothers who had participated in the original RCT were eligible candidates.

### 2.2. Study Procedure and Planning

For the present study, we approached women from the original RCT for participation approximately six years postpartum. Mothers and their children who consented visited the Florey Institute of Neuroscience and Mental Health in Melbourne between November 2016 and May 2017 for a cognitive assessment [26], a buccal swab donation to measure DNA methylation, and a series of Magnetic Resonance Imaging (MRI)-derived brain images of their child. All assessments were performed by radiographers and a researcher who were blinded to the treatment status of the women. The original RCT and the current follow-up study were both approved by the Human Research Ethics Committees of Austin Health, Melbourne, Australia. Trial Registration of the original RCT: ACTRN12607000397415. Registered on 2 August 2007. Informed consent was given by the children’s parents at the outset of the study. The study was conducted according to the declaration of Helsinki. Here, we describe the results of the MRI study. To facilitate the readability of the article, from now on we refer to the CBT group (children of mothers originally from the CBT group), and the TAU group (children of mothers originally from the TAU group).

### 2.3. MRI-Procedure

A head stabilizer was used, and headphones with extra padding around the ears were applied to reduce the noise exposure from the scanner. Children could watch a movie during the scanning procedure. The scans were performed on a 3T Siemens Skyra scanner (Erlangen Germany). We applied two scanning sequences, using “Multiband” as one of the parameters. Anatomical T1 weighted magnetization-prepared rapid acquisition gradient echo images (voxel size = 0.9 isotropic, repetition time = 1900 ms, inversion time = 950 ms, echo time = 2.5ms, flip angle = 10 degrees) and diffusion weighted images (63 directions, *b*-value = 2800 s/mm^2^, 2.5mm isotropic voxels, echo time = 100 ms, repetition time = 3940), were acquired. Scans were visually assessed for quality, and excluded from the analysis in case of substantial movement artefacts.

### 2.4. Characteristics

Baseline characteristics were extracted for both responders and non-responders to the current follow-up study from original study files. At follow-up, a sociodemographic questionnaire was again completed by the women, which included questions on current antidepressant medication, marital status, family income along with highest completed education and current symptoms of depression (BDI-II score) and anxiety (BAI score).

### 2.5. Statistical Analyses

#### 2.5.1. Voxel-Based Morphometry

Statistical Parametric Mapping (SPM8) (http://www.fil.ion.ucl.ac.uk/spm/) was used to process the high resolution T1 data. Firstly, the images were segmented to identify gray matter (GM), white matter (WM) and cerebrospinal fluid (CSF), using the “New Segment” tool in SPM8. The GM, WM and CSF segments for all subjects were registered using the well-established Diffeomorphic Anatomical Registration using Exponentiated Lie algebra (DARTEL) toolbox.

The ‘create warped’ tool then used the flow fields from the previous step to modulate and spatially normalize the GM, WM and CSF tissue segments towards the own space population template. 8mm smoothing by full width half maximum Gaussian kernel was applied to the modulated and spatially normalized images. Whole brain analyses were performed by *t*-tests on the smoothed, modulated and normalized GM images, with an absolute GM threshold of 0.2. To account for differences in brain volume, the estimated Total Intracranial Volume (eTIV) was used as a covariate-of-no-interest. This eTIV was calculated by combining the GM, WM, CSF segments from the subjects’ own space. We reported on voxels with an uncorrected *p* < 0.05 at cluster-level, and reported whether findings persisted after Family-Wise-Error (FWE) correction.

#### 2.5.2. Subcortical Gray Matter Volumes and Gray Matter Cortical Thickness 

Cortical reconstruction and volumetric segmentation was performed with the Freesurfer image analysis suite (http://surfer.nmr.mgh.harvard.edu/) [27,28]. Data were exported to SPSS version 23 for statistical analyses. Variables with a non-normal distribution were Log10 transformed. Regression analyses were performed with treatment allocation as an independent variable, and eTIV as a covariate. We used a threshold of *p* < 0.05 to indicate statistical significance in differences in gray matter volume or gray matter cortical thickness between the CBT and TAU groups. We additionally performed a Bonferroni correction to assess whether the results would persist after correction for the number of hypotheses tested. To assess whether a dose-response relationship existed between change in maternal mood symptoms during pregnancy and an offspring’s brain cortical thickness, regardless of treatment arm, we additionally created scatterplots to examine correlations between the change in symptom score of BDI-II (depression) and BAI (anxiety), from baseline to nine weeks post-randomization in the RCT in the whole sample with mean cortical thickness in both hemispheres, as well as with specific regions of interest, if an association with an offspring’s cortical thickness and maternal antenatal treatment status was shown in the initial analysis.

#### 2.5.3. Fixel-Based Analysis

Diffusion MRI connectivity analyses were performed using the Connectivity-Based Fixel Enhancement software [29], which was developed at the Florey Institute (Melbourne, Australia). Diffusion data was pre-processed, first by correcting for head motion, bias fields, and spatial up sampling by a factor of two. We performed global intensity normalization, by scaling all volumes by the median intensity of white matter voxels on *b* = 0 s/mm^2^ images. The fiber orientation distribution (FOD) was calculated using robust constrained spherical deconvolution [30] for each voxel, with a group average response function [31]. A study-specific FOD template was then created. FOD images for all participants were registered to the template. The amplitude of an FOD lobe is proportional to the volume of restricted water within axons of the given orientation, and can be interpreted as the apparent FD in that direction [31]. The FD was then calculated for each fixel, by integrating over the respective FOD lobe. Additional valuable information about tract morphology is available from the registration of each image to the template. By considering the local expansion or contraction of the warp field perpendicular to the fixel orientation, a relative measure of the FC is obtained [32]. Multiplying FD and FC at each fixel gives a combined measure of the fiber density and fiber bundle cross-sectional area (FDC) [32]. FDC is a more comprehensive measure of intra-axonal volume within a pathway, as it accounts for both microscopic axonal changes (detected as differences in diffusion signal), and macroscopic changes in a fiber-bundle (detected as relative differences in the registration warps). Statistical comparisons of FC, FD, and FDC between the CBT and TAU group were performed at each fixel, by one-way analysis of covariance (ANCOVA) with the contrasts of children from the CBT group versus children from the TAU group. We reported on fixels with an uncorrected *p* < 0.01, and reported whether findings persisted after FWE correction.

## 3. Results

### 3.1. Participants

**Figure 1:**
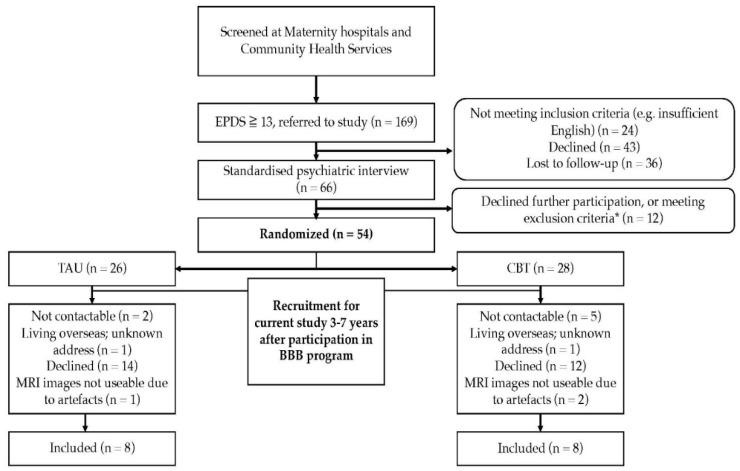
Flow diagram of participant recruitment. * Not meeting the inclusion criteria, comorbid axis I disorders, medical conditions at risk for interference with study participation, concurrent major psychiatric disorders for which the intervention was not designed (e.g., bipolar and psychotic disorder), risk requiring crisis management, current participation in other psychological programs, or significant difficulty with English.

Recruitment of participants is depicted in a flow-diagram (Figure 1). Of the 54 women who participated in the original study, 19 women agreed for their children to participate in the current follow-up study. MRI-scans of the children’s brains were conducted. The images of three children were not usable, due to substantial moving artefacts, resulting in eight children in both the CBT and TAU group, of whom useable data was available for the analyses. Table 1 shows baseline demographics of the non-responders and the responders to the current follow-up in the CBT and TAU groups, and Table 2 shows the current demographics of the participants. In line with CONSORT recommendations, no between-group significance tests were conducted on baseline values [33]. Visual inspection of the demographics indicated that, compared to the non-responders, the responders were overall more often born in Australia, with a higher income and educational level. Women who did not participate in the current follow-up had somewhat higher scores of depression and anxiety at baseline, compared to the women who did participate, which seemed more pronounced in the TAU group for depression, and more in the CBT group for anxiety (Table 1). Currently, women from the CBT group showed a somewhat higher income compared to women from the TAU group, and women from the TAU group more often used antidepressant medication (Table 2).

### 3.2. Voxel-Based Morphometry

**Figure 2:**
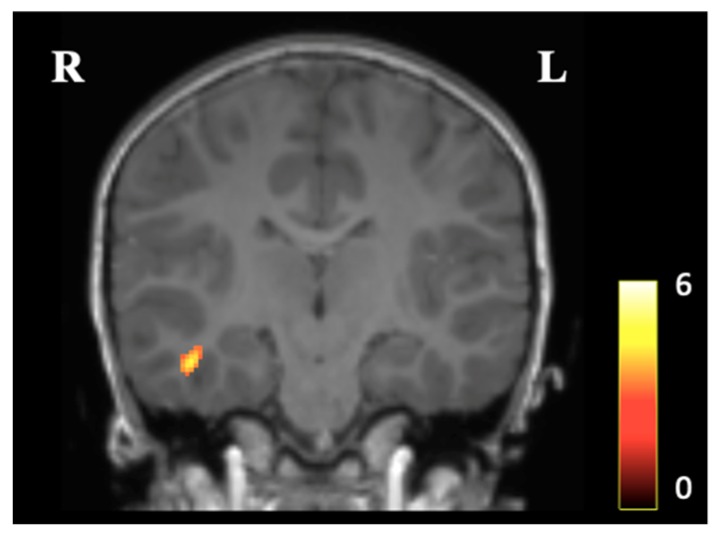
Presentation of the averaged VBM results in children aged approximately six years, in a longitudinal study built on a pilot Randomized Controlled Trial, comparing Cognitive Behavioral Therapy (CBT, *n* = 8) and Treatment as Usual (TAU, *n* = 8) for maternal antenatal depression treatment. The image shows increased gray matter concentration in the CBT group, compared to the TAU group, in the right medial temporal area at cluster level *p* < 0.05 uncorrected. The colored bar refers to Student’s *t*-score.

We identified one cluster showing larger gray matter concentration in the CBT compared to the TAU group at *p* < 0.05, which was located in the right medial temporal lobe (***K_E_*** = 201, *t* statistic = 4.36, *z* score (equiv.) = 3.36, Coordinates (x, y, z) = 45, −21, 21) (Figure 2). No voxels with increased gray matter concentration in the TAU group, compared to the CBT group, were identified. The analysis showed no statistically significant difference after FWE-correction (*p* = 0.415).

### 3.3. Subcortical Gray Matter Volumes and Gray Matter Cortical Thickness

**Figure 3:**
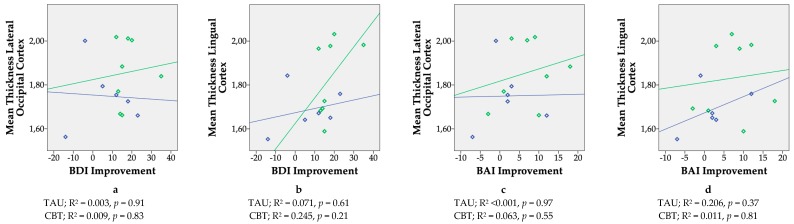
Scatterplots showing correlations between changes in maternal mood symptoms during pregnancy regardless of treatment status (defined as the difference in symptom score of the Beck Depression Inventory (BDI-II, a & b) and the Beck Anxiety Inventory (BAI, c & d) before and after treatment in a longitudinal study in children built on a pilot Randomized Controlled Trial, comparing maternal antenatal Cognitive Behavioral Therapy (CBT, *N* = 8) to Treatment as Usual (TAU, *N* = 6)) and cortical thickness of the children’s brain, approximately six years postpartum. Regions shown are those regions that showed increased cortical thickness in the children from the CBT relative to the TAU group (right hemisphere). X-axis: BDI = Beck Depression Inventory, BAI = Beck Anxiety Inventory. Higher scores indicate a better response to treatment. Y-axis: Mean Cortex Thickness Ratio = (mean cortex thickness region of interest (mm)/estimated Total Intracranial Volume(mm)); * 1,000,000. Green diamonds and slope = CBT, blue diamonds and slope = TAU.

**Figure 4:**
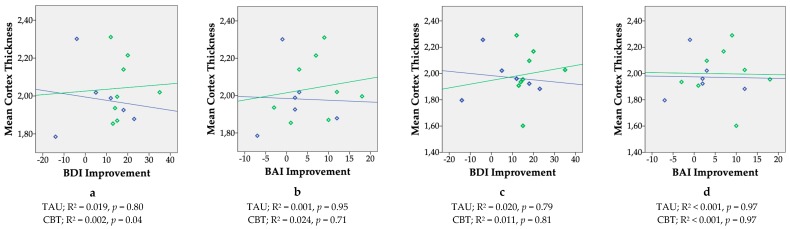
Scatterplots showing correlations between changes in maternal mood symptoms during pregnancy regardless of treatment status (defined as the difference in symptom score of the Beck Depression Inventory (BDI-II) and the Beck Anxiety Inventory (BAI) before and after treatment in a longitudinal study in children built on a pilot Randomized Controlled Trial, comparing maternal antenatal Cognitive Behavioral Therapy (CBT, *n* = 8) to Treatment as Usual (TAU, *n* = 6)) and cortical thickness of the children’s brain, approximately six years postpartum. Regions shown are the mean cortex thicknesses of the left (a & b) and right (c & d) hemisphere. X-axis: BDI = Beck Depression Inventory, BAI = Beck Anxiety Inventory. Higher scores indicate a better response to treatment. Y-axis: Mean Cortex Thickness Ratio = ((mean average cortex thickness (mm))/estimated Total Intracranial Volume(mm)) * 1,000,000. Green diamonds and slope = CBT, blue diamonds and slope = TAU.

Children from the CBT group had a thicker lateral occipital cortex (mean difference = 0.13 mm, 95% CI = 0.005 to 0.26) and lingual gyrus (mean difference = 0.18 mm, 95% CI = 0.01 to 0.34) in the right hemisphere at *p* < 0.05 (Table 3). After applying a Bonferroni correction for the number of hypotheses tested (α = 0.05/34 = 0.0015), both differences were no longer statistically significant. Children from the CBT group did not differ from children from the TAU group in terms of cortical thickness in any of the regions in the left hemisphere (Table 4). Figure 3 shows scatterplots of the absolute change in symptom score of BDI-II (depression) and BAI (anxiety) regardless of treatment, nine weeks after randomization in the RCT, as a function of mean cortical thickness of the right lateral occipital cortex and right lingual cortex. Figure 4 shows scatterplots of the absolute change in symptom score of BDI-II (depression) and BAI (anxiety) regardless of treatment, nine weeks after randomization in the RCT, as a function of mean cortical thickness across the whole brain. Due to incomplete BDI-II and BAI post-treatment scores of two women in the TAU group, the results here are shown for eight children from the CBT and six children from the TAU group. Most plots revealed positive slopes for the CBT group, but none were statistically significant. No statistically significant differences were observed between the CBT and TAU group’s mean subcortical volumes (Table 5).

### 3.4. Fixel-Based Diffusion

**Figure 5:**
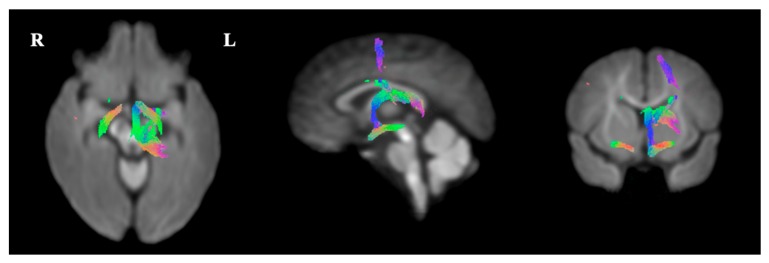
Presentation of the FBA results, indicating white matter pathways, showing increased fiber-bundle cross-section in the TAU group, compared to the CBT group at *p* < 0.01 uncorrected, in children aged approximately six years, in a longitudinal study built on a pilot Randomized Controlled Trial comparing Cognitive Behavioral Therapy (CBT, *n* = 8) and Treatment as Usual (TAU, *n* = 8) for maternal antenatal depression. To enable the visualization of the fixels in 3D, streamlines from the template-derived whole-brain tractogram were ‘cropped’ to include streamline points that correspond to fixels, and colored by direction (red: Left-right, blue: Inferior-superior, green: Anterior-posterior).

**Figure 6:**
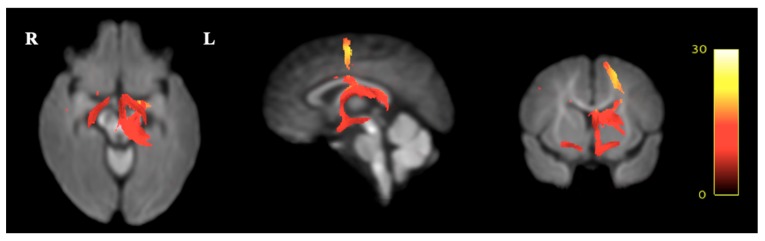
Presentation of the FBA results expressing the effect sizes as a relative increase (from red to yellow, indicating 0% to 30% difference) in fiber-bundle cross-sections in the TAU group relative to the CBT group, in children aged approximately six years, in a longitudinal study built on a pilot Randomized Controlled Trial comparing Cognitive Behavioral Therapy (CBT, *n* = 8) and Treatment as Usual (TAU, *n* = 8) for maternal antenatal depression. The Fornix and Optic Tract showed an increase of 10–20%, and the Stria Terminalis showed an increase of 20–25% in fiber-bundle cross-section in the TAU group compared to the CBT group.

In the CBT group, decreased white matter FC was seen in fixels along white matter tracts, corresponding to the location and shape of the Fornix, the Stria Terminals and the Optical Tract, compared to the TAU group at *p* < 0.01 (Figure 5). Relative to the TAU group, the CBT group showed a decrease in FC in the Fornix and Optic Tract of 10–20%, and in the Stria Terminalis of 20–25% (Figure 6). However, no differences were seen in FD or in the combined FDC measure between both groups that corresponded to a white matter tract. Again, none of the analyses showed statistically significant differences after FWE-correction.

## 4. Discussion

We performed a longitudinal brain MRI study in 6-year-old children, built on a prior pilot RCT comparing Cognitive Behavioral Therapy (CBT) and Treatment as Usual (TAU) for maternal antenatal depression. We found no robust evidence for a treatment effect of CBT for antenatal depression on (micro)structural brain characteristics in 6-year-old offspring. Results from exploratory analyses (refraining from adjusting the results for multiple testing), indicated that children born to women receiving CBT for antenatal depression exhibited a cluster with increased gray matter concentration in the right medial temporal lobe, as well as a thicker lateral occipital cortex and lingual gyrus in the right hemisphere, compared to the TAU group. An unexpected finding was smaller FC along white matter tracts corresponding to the Fornix, the Stria Terminalis and the Optical Tract in the children from the CBT group. Although none of the results survived correction for multiple testing, and the sample size was extremely small, most trends we observed in exploratory analyses were in the hypothesized direction. Antenatal CBT for depression thus may possibly ameliorate offspring brain (micro)structural outcomes, but substantially larger studies are warranted to confirm our preliminary conclusions that CBT for antenatal depression affects brain development in children.

Growing evidence suggests that the origins of neurodevelopmental disorders can be traced back to the intrauterine period of life, during sensitive periods of cellular proliferation, differentiation and maturation [4]. In animal models, changes in brain morphology have been observed in offspring of mothers exposed to prenatal stress, such as expanded dimensions of the lateral nucleus of the amygdala [34], and reduced spine density and dendritic complexity in the prefrontal cortex [35] in prenatally stressed animals. In our exploratory analyses, we observed higher gray matter concentration in the CBT group, compared to the TAU group in the right medial temporal lobe. This is in line with earlier studies showing associations between prenatal stress, anxiety, and depression with decreased gray matter volume and cortical thinning, in (among other regions), the temporal lobe [15,16,36,37]. The medial temporal lobe is part of multiple brain networks that have been shown to play an important role in processes related to auditory language processing and language learning in children [38,39]. Also, there was a trend towards a thicker lateral occipital cortex and lingual gyrus in the right hemisphere in the CBT group. Cortical thinning in regions associated with cognitive performance has been a consistent finding in studies investigating the effect of prenatal exposure to maternal stress, depression, anxiety or cortisol on neurodevelopment [15,17,40]. Although the observed differences were small, and not robust after correction for multiple testing, our results imply that treatment of depression in pregnancy may potentially modify the negative direction of the associations described between prenatal exposure to maternal depression and an offspring’s cortical thickness [15,16,41]. In literature, there is some evidence for a dose-response effect of prenatal stress and anxiety on child neurodevelopment [8,42]. We therefore additionally examined whether improvement of symptom scores in depression or anxiety directly after treatment correlated with cortical thickness of the lateral occipital cortex and lingual gyrus in the right hemisphere, as well as across the whole brain. We did not find evidence for any statistically significant correlation. The slopes in most figures showed small positive tendencies in the CBT group for depression and anxiety improvement and cortical thickness, potentially indicating a subtle dose-response trend between absolute improvement in depression and anxiety symptom score, and increased cortical thickness of the offspring brain, although this clearly requires replication in larger study samples. We found no differences in volumes of the amygdala, hippocampus, and cerebellum between the CBT and TAU groups. We had expected to find volumetric differences of mainly the amygdala, a structure that plays a pivotal role in emotional behavior, and is closely associated with stress reactivity and vulnerability for depressive disorder [43,44,45]. In one study of similar size (*n* = 19), children exposed to clinically relevant maternal depressive symptoms during pregnancy showed increased amygdala responses to negative emotional faces, compared to control children [46]. Moreover, higher amygdala volume has been linked to prenatal depression and maternal cortisol levels in earlier studies [18,47].

Nevertheless, our findings correspond with a large observational study that examined the association between prenatal depressive symptoms and child brain morphology at the age of six years, and could not detect an association with amygdala volume either [17].

An unexpected finding in contrast to our hypothesis was smaller white matter FC in children from the CBT group along the tracts of the Fornix, the Stria Terminalis and the Optical tract. The Stria Terminalis is involved in a range of behaviors that are implicated in psychiatric disease, including the stress response [48], extended duration fear states [49] and social behavior [50]. Our results therefore imply that untreated antenatal depression may lead to increased white matter fiber FC in white matter tracts in the offspring that play a role in the development of psychopathology in later life. As far as we are aware, fixel-based analysis has not yet been studied in a developmental context which complicates comparisons with prior study results, in which fractional anisotropy (FA) is commonly used as an estimate for white matter microstructure. FA indicates the coherence of brain fiber organization, although this has shown to be only a crude derivative of actual individual fiber anisotropy [51]. A prior study reported lower FA in the amygdala of neonates prenatally exposed to high maternal depressive symptoms. [14]. However, in a similar study, infants born to mothers with higher prenatal maternal depressive symptoms, showed greater functional connectivity of the amygdala with other brain regions, mimicking patterns of connectivity observed in adolescents and adults with major depressive disorder [13]. Another study found a *positive* association between white matter integrity (defined as increased FA) in neonates and prenatal exposure to maternal depressive symptoms, although this was restricted to the uncinate fasciculus in boys [52]. The authors propose that their results, which at a first glance seem counter-intuitive, may actually be indicative of accelerated brain maturation after prenatal depression exposure, with potentially increased risk for psychopathology in later life [53]. Although speculative, smaller white matter FC of the Fornix and Stria Terminalis in children of mothers treated for antenatal depression may indicate lower functional connectivity and hence a lower response to stress, but this evidently requires further examination in future studies. As far as we are aware, the effects of prenatal depression on Optic Tract white matter integrity have not been studied, but greater FA in the left optic radiation has been associated with bipolar disorders in an earlier study, indicating that microstructural alterations in this particular tract may correlate to certain psychopathologic disorders [54].

This study has some important limitations that need to be considered when interpreting the results. The most important limitation is that of the small sample size, indicating very low power to detect relevant effect sizes. Therefore, the results from this study should be interpreted with great caution. Only 14.8% of all women and their children were willing to participate in the current MRI study. This highlights the major challenge of performing long-term follow-ups of intervention studies for antenatal depression that include biological assessments in children. Nevertheless, these studies are vital, as they are currently lacking, and can provide unique evidence on whether psychological treatment for depression during pregnancy may affect offspring brain development [2]. We emphasize that our results should be considered as explorative, and can be used for the design of sufficiently powered future trials. Also, the analyses were performed without the inclusion of covariates, since the CBT and TAU groups were reasonably balanced in terms of mother and child characteristics. Because of the relatively broad age range of children, we compared our unadjusted results with the results including child age as an additional covariate. This did not substantially alter our results, and therefore the unadjusted results were reported. However, we cannot rule out that confounding has occurred due to (unmeasured) postnatal experiences as a result of improved maternal mood in pregnancy, such as better mother-infant attachment, or more positive parenting [55]. This may be reflected by the observation that women from the TAU group more often used antidepressant medication during the follow-up, which potentially has impacted the outcomes through parenting by a more depressed mother. Nevertheless, despite the higher rate of antidepressant use in the TAU group, symptom scores of maternal depression and anxiety during the follow-up were about similar in both groups, indicating that differences in exposure to postnatal maternal mood complaints and associated behavioral parental changes between the two groups has been limited.

Finally, it should be noted that the responders had a higher income, and were more highly educated compared to non-responders. They also reported lower scores of depression and anxiety at baseline, and a smaller reduction in symptoms after treatment. The less apparent effectiveness of treatment in the responders compared to the non-responders may have led to an underestimation of the true effects of antenatal depression treatment on offspring brain morphology and connectivity.

## 5. Conclusions

We describe, for the first time, brain MRI findings in 6-year old children in a longitudinal study that was built on a pilot RCT on the effectiveness of CBT for maternal antenatal depression. We found no robust evidence for a treatment effect of CBT for antenatal depression on (micro)structural brain characteristics in 6-year-old offspring. Results from exploratory analyses (refraining from adjusting the results for multiple testing) indicated that antenatal CBT may increase cortical thickness and gray matter volume, and may also reduce fiber-bundles cross-section of white matter tracts involved in the stress-response in the 6-year-old offspring. However, the effects were not statistically significant after correcting for multiple testing, and the sample size was extremely small. We emphasize that our results should therefore be interpreted with great caution, and considered as explorative. Substantially larger studies are warranted before we can confirm or refute the hypothesis that CBT during pregnancy affects child brain development. This will have important implications for clinicians who are confronted with pregnant women with depression and anxiety disorders, as it will show whether timely detection and treatment of antenatal depression will not only benefit the mother’s mental well-being, but that of her child also.

## Figures and Tables

**Table 1:** Baseline maternal demographics of responders and non-responders of a longitudinal study approximately six years after participation in a pilot Randomized Controlled Trial comparing Cognitive Behavioral Therapy (CBT) and Treatment as Usual (TAU) for antenatal depression.

Baseline Demographics	Non-Responders		Responders	
	CBT (*n* = 20)	TAU (*n* = 18)	CBT (*n* = 8)	TAU (*n* = 8)
BDI-II pre-treatment score	31.8 ± 8.6	32.5 ± 7.8	29.8 ± 11.3	26.4 ± 10.1
BAI pre-treatment score	25.0 ± 10.5	23.1 ± 11.2	18.8 ± 9.7	17.5 ± 6.4
BDI-II post-treatment score ^1^	13.7 ± 9.4	18.4 ± 11.3	12.0 ± 10.7	18.3 ± 6.2
BAI post-treatment score ^1^	11.1 ± 7.0	17.4 ± 12.9	11.6 ± 10.9	15.5 ± 8.0
∆ BDI-II score (pre-treatment–post-treatment)	18.9 ± 10.9	15.0 ± 12.0	17.8 ± 7.4	6.7 ± 13.9
∆ BAI score (pre-treatment–post-treatment)	13.5 ± 9.7	5.5 ± 8.8	7.1 ± 6.7	1.8 ± 6.2
Gestational age at enrolment in weeks	21.3 ± 7.6	21.4 ± 6.5	16.6 ± 7.2	20.0 ± 5.1
Antidepressant use at baseline (%)	15.8	27.8	0	12.5
Marital status (%)				
Married	60.0	66.7	62.5	62.5
De Facto	35.0	16.7	25	25
Separated	0	5.6	0	12.5
Single	5.0	11.1	12.5	0
Birth location (%)				
Australia	70.0	66.7	87.5	100
Other	30.0	23.3	12.5	0
Income (%)				
Up to $20,000	5.3	5.9	0	12.5
$20,001−$40,000	5.3	23.5	12.5	12.5
$40,001−$60,000	26.3	23.5	0	0
$60,001−$80,000	21.1	23.5	37.5	25
>$80,001	31.6	17.6	37.5	50
Do not wish to divulge	10.5	5.9	12.5	0
Highest level of education (%)				
Did not finish school	5.0	16.7	0	0
High School	10.0	22.2	0	25
Certificate Level/Apprenticeship	25.0	0	12.5	12.5
Advanced Diploma	15.0	11.1	25	0
Bachelor degree	25.0	16.7	0	25
Graduate diploma/certificate	10.0	16.7	37.5	25
Postgraduate Degree	10.0	16.7	25	12.5

Notes: Data are given as means ± SD, or in percentages in case of dichotomous or categorical outcomes. ^1^: The Beck Depression Inventories (BDI)-II and Beck Anxiety Inventories (BAI) post-treatment scores were available for 8/8 women from the CBT, and 6/8 women from the TAU group.

**Table 2:** Current demographics of women and their children approximately six years after antenatal depression treatment in a pilot Randomized Controlled Trial comparing Cognitive Behavioral Therapy (CBT) and Treatment as Usual (TAU) for antenatal depression.

Women	CBT (*n* = 8)	TAU (*n* = 8)
Age at giving birth	34.1 ± 6.0	35.6 ± 3.9
Current BDI-II	15.0 ± 15.8	14.4 ± 10.1
Baseline BDI-II	29.8 ± 11.3	26.4 ± 10.1
Current BAI	11.1 ± 9.5	10.4 ± 11.3
Baseline BAI	18.8 ± 9.7	17.5 ± 6.4
Antidepressant use (%)	12.5	50
Marital status (%)		
Married	62.5	75
De Facto	12.5	12.5
Separated/Single	25	12.5
Family Income (%)		
Up to $20,000	0	12.5
$20,0001–$40,000	12.5	0
$40,001–$60,000	0	12.5
$60,001–$80,000	0	12.5
>$80,001	87.5	62.5
Highest level of education (%)		
Did not finish school	0	0
High school	0	25
Certificate level/apprenticeship	12.5	12.5
Advanced diploma	0	0
Bachelor degree	12.5	12.5
Graduate diploma/certificate	50	12.5
Postgraduate Degree	25	37.5
Children		
Age (years)	5.7 ± 1.0	6.2 ± 0.8
Birth weight (grams)	3622 ± 343	3393 ± 580
Gender (boys) (%)	62.5	75

Note: Data are given as means ± SD, or in percentages in case of dichotomous or categorical outcomes.

**Table 3:** Average Magnetic Resonance Imaging (MRI)-derived local gray matter thickness differences in the right hemisphere in children aged approximately six years in a longitudinal study, built on a pilot Randomized Controlled Trial comparing Cognitive Behavioral Therapy (CBT) and Treatment as Usual (TAU) for maternal antenatal depression.

Region of Interest	CBT (*n* = 8)	TAU (*n* = 8)	Regression Coefficients ^1^	
	Mean mm ± SD	Mean mm ± SD	*B*	95% CI
Superior Frontal Gyrus	2.93 ± 0.24	3.02 ± 0.17	−0.07	−0.32 to 0.17
Caudal Middle Frontal Cortex	2.72 ± 0.15	2.79 ± 0.21	−0.06	−0.27 to 0.15
Rostral Middle Frontal Gyrus	2.73 ± 0.22	2.77 ± 0.18	−0.04	−0.28 to 0.20
Inferior Frontal Gyrus–Pars Opercularis	2.96 ± 0.22	2.96 ± 0.13	0.02	−0.19 to 0.23
Inferior Frontal Gyrus–Pars Orbitalis	3.09 ± 0.12	3.14 ± 0.25	−0.05	−0.28 to 0.18
Inferior Frontal Gyrus–Pars Triangularis	3.00 ± 0.17	2.96 ± 0.12	0.05	−0.12 to 0.22
Precentral Gyrus	2.60 ± 0.26	2.61 ± 0.20	0.02	−0.24 to 0.28
Postcentral Gyrus	2.27 ± 0.22	2.32 ± 0.19	−0.06	−0.30 to 0.18
Supramarginal Gyrus	2.79 ± 0.22	2.68 ± 0.21	−0.11	−0.36 to 0.14
Superior Temporal Gyrus	3.06 ± 0.18	3.04 ± 0.20	0.03	−0.19 to 0.24
Middle Temporal Gyrus	3.13 ± 0.17	3.10 ± 0.14	0.05	−0.12 to 0.22
Inferior Temporal gyrus	3.13 ± 0.15	2.99 ± 0.18	0.16	−0.02 to 0.35
Fusiform Gyrus	3.01 ± 0.09	3.0 ± 0.15	0.02	−0.12 to 0.16
Lateral Occipital Cortex	2.59 ± 0.12	2.48 ± 0.13	0.13	0.005 to 0.26 *
Lingual Gyrus	2.55 ± 0.11	2.40 ± 0.18	0.18	0.01 to 0.34 *
Cuneus	2.38 ± 0.09	2.38 ± 0.14	0.02	−0.12 to 0.15
Insular Cortex ^2^	3.37 ± 0.19	3.4 ± 0.14	−0.004	−0.03 to 0.02

^1^ Covariate included in the analyses: Estimated Total Intracranial Volume; ^2^ Log10 transformed; * *p* < 0.05.

**Table 4:** Average MRI-derived Local gray matter thickness differences in the left hemisphere in children aged approximately six years in a longitudinal study, built on a pilot Randomized Controlled Trial, comparing Cognitive Behavioral Therapy (CBT) and Treatment as Usual (TAU) for maternal antenatal depression.

Region of Interest	CBT (*n* = 8)	TAU (*n* = 8)	Regression Coefficients ^1^	
	Mean mm ± SD	Mean mm ± SD	*B*	95% CI
Superior Frontal Gyrus	2.97 ± 0.17	3.06 ± 0.15	−0.08	−0.26 to 0.10
Caudal Middle Frontal Cortex	2.65 ± 0.26	2.81 ± 0.17	−0.15	−0.41 to 0.10
Rostral Middle Frontal Cortex	2.82 ± 0.17	2.83 ± 0.19	0.001	−0.21 to 0.21
Inferior Frontal Gyrus–Pars Opercularis	3.06 ± 0.17	3.03 ± 0.18	0.05	−0.15 to 0.25
Inferior Frontal Gyrus–Pars Orbitalis ^2^	3.29 ± 0.25	3.15 ± 0.17	0.02	−0.02 to 0.05
Inferior Frontal Gyrus–Pars Triangularis	3.05 ± 0.16	2.9 ± 0.19	0.14	−0.05 to 0.34
Precentral Gyrus	2.67 ± 0.19	2.67 ± 0.13	−0.002	−0.19 to 0.18
Postcentral Gyrus ^2^	2.36 ± 0.13	2.28 ± 0.13	0.11	−0.01 to 0.24
Supramarginal Gyrus	2.90 ± 0.23	2.77 ± 0.23	0.18	−0.07 to 0.42
Superior Temporal Gyrus	3.11 ± 0.25	3.01 ± 0.13	0.11	−0.12 to 0.34
Middle Temporal Gyrus ^2^	3.17 ± 0.08	3.08 ± 0.13	0.11	−0.01 to 0.22
Inferior Temporal Lobe	3.19 ± 0.13	3.13 ± 0.19	0.11	−0.05 to 0.26
Fusiform Gyrus ^2^	3.02 ± 0.10	3.0 ± 0.13	0.007	−0.01 to 0.02
Lateral Occipital Cortex	2.56 ± 0.11	2.46 ± 0.11	0.12	−0.01 to 0.25
Lingual Gyrus	2.53 ± 0.20	2.43 ± 0.23	0.11	−0.14 to 0.36
Cuneus	2.48 ± 0.16	2.43 ± 0.19	0.08	−0.15 to 0.27
Insular cortex	3.43 ± 0.15	3.44 ± 0.21	−0.02	−0.23 to 0.19

^1^ Covariate included in the analyses: Estimated Total Intracranial Volume; ^2^ Log10 transformed.

**Table 5:** Subcortical volumetric differences in children aged approximately six years, in a longitudinal study built on a pilot Randomized Controlled Trial, comparing Cognitive Behavioral Therapy (CBT) and Treatment as Usual (TAU) for maternal antenatal depression.

Subcortical Structure	CBT (*n* = 8)	TAU (*n* = 8)	Regression Coefficients ^1^	
	Mean mm^3^ ± SD	Mean mm^3^ ± SD	*B*	95% CI
Total Gray Matter	804,176 ± 52,916	82,706 ± 74,533	4140	−33,620 to 41,900
Total White Matter	404,846 ± 24,130	434,449 ± 55,768	−13,482	−44,932 to 17,969
Amygdala	3150 ± 257	3331 ± 331	−98	−378 to 182
Cerebellum	115,228 ± 11,175	122,146 ± 109,478	−4667	−16,290 to 6955
Hippocampus	8042 ± 794	8391 ± 652	−110	−728 to 509

^1^ Covariate included in the analyses: Estimated Total Intracranial Volume.
